# A Cautionary Tale: Urticaria and Angioedema As Presenting Signs of Lymphoproliferative Disorders

**DOI:** 10.7759/cureus.106738

**Published:** 2026-04-09

**Authors:** Dariush Kafashzadeh, Rita Kachru

**Affiliations:** 1 Allergy and Immunology, University of California Los Angeles, Los Angeles, USA

**Keywords:** acquired, angioedema, lymphoma, lymphoproliferative, malignancy, urticaria

## Abstract

Urticaria and angioedema are common conditions evaluated by allergists. Still, their potential association with lymphoproliferative and myeloproliferative conditions underscores the importance of screening for red-flag syndromes and performing an appropriate workup. Here, we present the case of a 57-year-old woman who presented with seemingly idiopathic angioedema and urticaria that was partially responsive to standard antihistamine therapy but was found to have an underlying malignancy and whose symptoms greatly improved with treatment of the driving condition.

## Introduction

Acquired angioedema (AAE) due to C1 inhibitor deficiency is a rare syndrome that presents after middle age with recurrent swelling of the skin, gastrointestinal tract, and upper respiratory tract [1]. This condition is strongly associated with lymphoproliferative disorders, ranging from monoclonal gammopathy of uncertain significance (MGUS) to non-Hodgkin lymphoma [2]. The pathophysiology involves excessive C1 inhibitor (C1-INH) catabolism, occurring through two mechanisms: direct consumption of C1-INH by neoplastic lymphatic tissue, with continuous activation of the classical complement pathway, or the production of anti-C1-INH autoantibodies that promote ineffective C1-INH-protease interactions [1]. Reduced C1-INH function leads to bradykinin generation, which increases vascular permeability and induces angioedema. Diagnosis is confirmed by low C1-INH levels and function and low C1q (distinguishing it from hereditary angioedema) [1]. Treatment focuses on both managing acute angioedema episodes with bradykinin-targeting therapies and, just as critically, addressing the underlying lymphoproliferative disorder, which can lead to improvement or remission of the angioedema. Urticaria can also, albeit rarely, be associated with malignancies, such as in Schnitzler syndrome. This syndrome is characterized by chronic urticaria and monoclonal IgM gammopathy, and patients may progress to lymphoplasmacytic neoplasia, particularly Waldenström macroglobulinemia or systemic marginal zone B-cell lymphoma [3].

Here, we present the case of a 57-year-old woman who presented with late-onset angioedema with abdominal pain and abnormal complement studies and was ultimately found to have an acquired C1-inhibitor deficiency associated with an underlying malignancy.

## Case presentation

The patient is a 57-year-old woman who initially presented with urticaria, angioedema, and abdominal pain. Patient was without other atopic history or recurrent infections. The patient had a remote history of intermittent pruritic hives, but these had worsened in the last two years. Hives temporarily resolved with cetrizine 10 mg twice a day and famotidine 20 mg twice a day. Still, the patient had also developed, in the last year, intermittent episodes of stabbing abdominal pain lasting between one and five days, as well as self-reported visible neck swelling. Physical exam notable for mild splenomegaly.

The patient’s relevant lab workup is shown in Table 1. She was found to have hereditary alpha-tryptasemia, hypocomplementemia, IgM kappa monoclonal gammopathy, and lymphopenia. Given that a low C1q is pathognomonic for AAE, the patient was urgently referred to hematology/oncology to rule out myeloproliferative/lymphoproliferative processes such as mastocytosis, lymphoma, or multiple myeloma.

**Table 1:** Relevant lab findings μL: microliter, mg: milligram, dL: deciliter, mcg: microgram, mL: milliliter, %: percent, g: gram, L: liter

Lab	Value	Normal range (units)
Absolute lymphocyte count	1220	1300-3400 (cells/μL)
C4	<2	15-57 (mg/dL)
C1q	<50	109-242 (mcg/mL)
C1 esterase inhibitor	<8	21-38 (mg/dL)
C1 esterase inhibitor function	15	≥41 (%)
Serum monoclonal protein	0.2	0 (g/dL)
Serum kappa quantitative free light chains	56.7	3.3-19.4 (mg/L)
Serum lambda quantitative free light chains	18.7	5.7-26.3 (mg/L)
TPSAB1	3 alpha-tryptase copies 2 beta-tryptase copies	2 alpha-tryptase copies with 2 beta-tryptase copies (of the TPSAB1 gene, respectively)
Tryptase	18	<11 (mcg/L)

**Figure 1:**
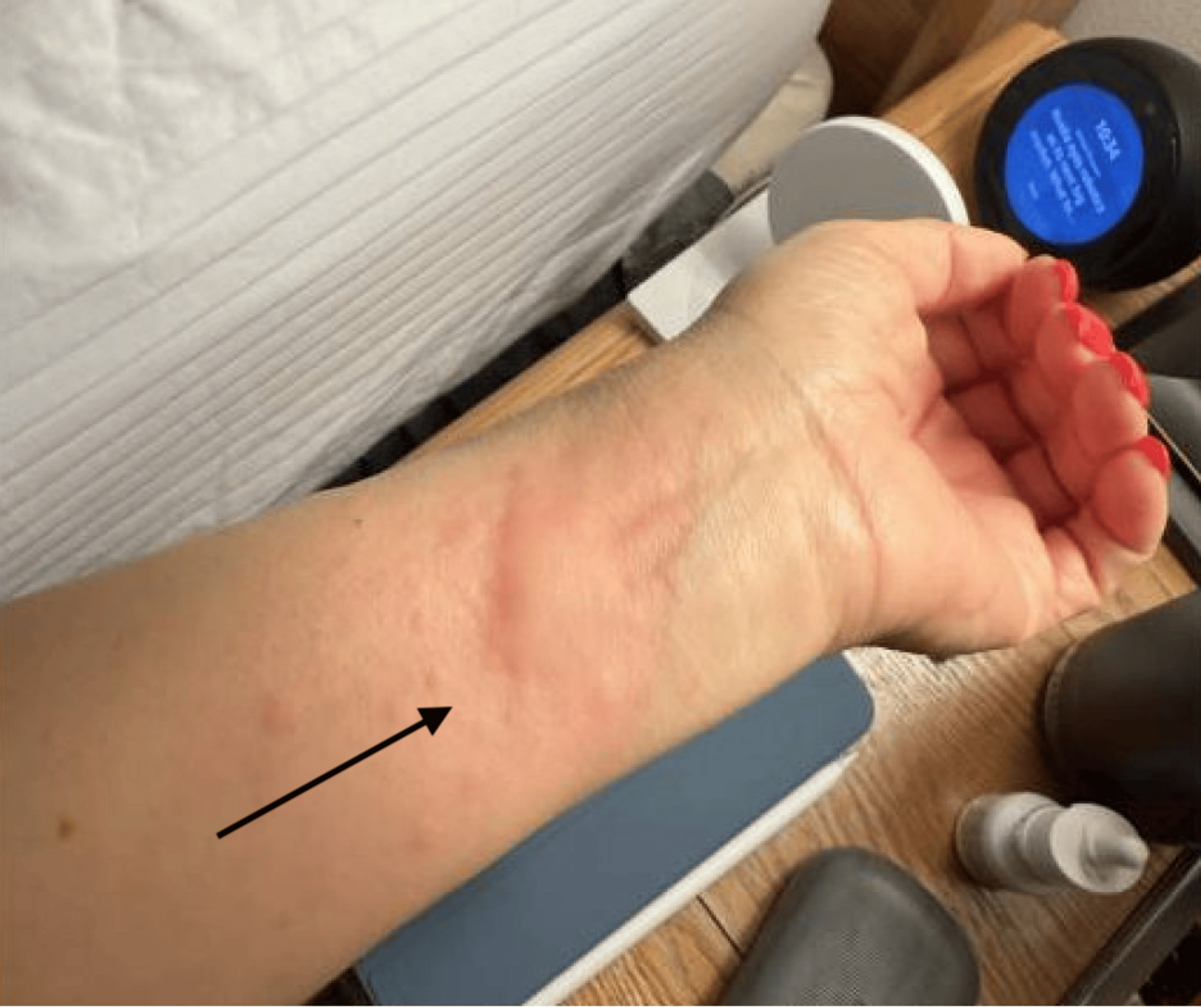
Recurrent urticaria Urticaria on the patient's arm that had recurred despite being on antihistamine therapy. The black arrow highlights the prominent hives the patient was experiencing.

The patient’s urticaria had returned and persisted at this point, as seen in Figure 1. The patient's C-Kit mutation was negative, and the initial plan was to start the patient on omalizumab for refractory urticaria; however, a bone marrow biopsy was eventually diagnostic of low-grade B-cell lymphoma involving 15% of the marrow (marginal zone lymphoma vs. Waldenstrom macroglobulinemia). The sequencing panel showed mutations in CARD11, KLF2, and NOTCH1 and a normal karyotype/FISH. Patient subsequently underwent chemotherapy with bendamustine and rituximab. Since her second round of chemotherapy, urticaria, angioedema, and abdominal pain have resolved with decreased antihistamine use (despite underlying hereditary alpha-tryptasemia), with plans to further wean off.

## Discussion

While hives and angioedema are rarely the presenting symptoms of lymphoproliferative or myelodysplastic syndromes, there is a recognized association that warrants consideration in specific clinical contexts. The frequency is low, and current guidelines generally do not recommend routine screening for malignancy in patients with chronic urticaria unless specific clinical features suggest an underlying hematologic disorder [4].

In a large population-based cohort study of patients with chronic urticaria, there was a significantly elevated risk for hematologic malignancies with a standardized incidence ratio of 4.1 (95% CI, 3.1-5.4), particularly for non-Hodgkin lymphoma (SIR 4.4) and leukemia (SIR 3.7) [5]. However, the absolute numbers remain small. Among 12,720 patients with chronic urticaria, only 58 developed hematologic malignancies over an eight-year follow-up period [5]. The risk was highest in the first year after diagnosis [5].

The most clinically important association is AAE due to C1-inhibitor deficiency, which is strongly linked to lymphoproliferative disorders, as was the case in this patient. Approximately 46% of patients with AAE have an underlying hematologic disorder, including MGUS or B-cell malignancies [2]. Importantly, angioedema may precede the development of overt malignancy by several years, necessitating annual evaluation if no underlying malignancy is initially found [6].

Schnitzler syndrome represents another critical entity characterized by chronic urticaria (usually nonpruritic), monoclonal IgM gammopathy, and systemic symptoms including fever, bone pain, and arthralgia. While classified as an autoinflammatory disorder, it carries a long-term risk of progression to lymphoproliferative disease, particularly Waldenström macroglobulinemia [3].

Both generalists and allergists typically do not routinely work up chronic urticaria for underlying malignancy unless specific red flags are present. A retrospective review of 1,872 laboratory tests in 356 patients with chronic urticaria found that extensive testing led to a change in management in only one patient (0.3%) [7].

The recommended approach includes a comprehensive history and physical examination, with limited basic testing (CBC with differential, ESR, or CRP) to exclude differential diagnoses [4]. Further investigation for malignancy should be pursued only when suggested by clinical features such as systemic symptoms (fever, weight loss, night sweats); late-onset symptoms (>60 years); antihistamine-refractory disease; wheals lasting >24 hours with post-inflammatory hyperpigmentation; isolated angioedema refractory to antihistamines and corticosteroids; elevated inflammatory markers; abnormal blood counts; monoclonal gammopathy on serum protein electrophoresis; or low C4 and C1q levels (suggesting acquired C1-inhibitor deficiency) [8].

## Conclusions

The case we discuss here underscores the importance of a thorough history and awareness of potential connections to more serious conditions when patients present with relatively common conditions such as hives and angioedema. The above guidelines are evidence-based, but many clinical features warrant further lab workup to rule out hematological disease and are often overlooked and underdiscussed, with potentially serious consequences. While allergists are the main providers caring for these conditions, generalists are often the first to see these patients. They are involved in initial treatment and workup; thus, increasing their awareness of these potential clinical associations and red-flag signs is critically important.
